# Analyzing Active Constituents and Optimal Steaming Conditions Related to the Hematopoietic Effect of Steamed *Panax notoginseng* by Network Pharmacology Coupled with Response Surface Methodology

**DOI:** 10.1155/2020/9371426

**Published:** 2020-07-26

**Authors:** Shuhan Zhou, Nan Jiang, Min Zhang, Xiao Xiao, Zhiyi Liu, Xiaohui Xu, Qinghua Gao, Wenliang Lv

**Affiliations:** ^1^Clinical College, Hubei University of Chinese Medicine, Wuhan 430000, China; ^2^Lab Animal Research Center, Hubei Hospital of Traditional Chinese Medicine, Wuhan, China; ^3^Hubei Academy of Traditional Chinese Medicine, Wuhan, China; ^4^Lab Animal Research Center, Hubei University of Chinese Medicine, Wuhan 430000, China; ^5^College of Basic Medicine, Hubei University of Chinese Medicine, Wuhan 430000, China

## Abstract

During hundreds of years of medication, it is believed that the steamed *Panax notoginseng* (SPN) can enrich and regulate the blood, replenish the body, and improve the health. The aim of this study was to optimize the steaming conditions of SPN which are related to the hematopoietic effect. In the study, network pharmacology and pharmacological experiments were used to predict and verify the potential hematopoietic active ingredients of SPN. Three variables including the steaming time (2-10 h), steaming temperature (90-130°C), and different producing areas of PN were investigated by using single-factor analysis. Box-Behnken design response surface methodology (BBD-RSM) was performed to explore the optimized steaming conditions which are responsible for the hematopoietic effect of SPN. Furthermore, the hematopoietic effect of the optimized SPN was evaluated. Results demonstrated that ginsenoside Rd, Rh_1_, Rh_4_, Rk_3_, and 20(*S*)-Rg_3_ can significantly increase blood routine parameters and expressions of hematopoietic factors in anemia mice. The total contents of the five ginsenosides were selected as evaluation indexes of the response surface method. We found that the PN from Wenshan steamed at 120°C for 5 h could significantly increase the levels of blood routine parameters and hematopoietic factor expression compared with the model group. The study not only provides data support for the determination of hematinic effect-related markers for SPN but also gives a scientific reference for the processing of SPN which has a better hematopoietic effect. The underlying mechanisms require further research.

## 1. Introduction

During hundreds of years of medication, there was a description for *Panax notoginseng* (PN) properties that “the raw PN (RPN) materials eliminate and the steamed ones tonify” [1, 2]. The so-called “eliminate” means that the RPN can stop bleeding, eliminate blood stasis, promote blood circulation, diminish swelling, and ease pain. The “tonify” means that the steamed PN (SPN) can enrich and regulate the blood, replenish the body, and improve the health. Prevailing studies confirmed that the RPN has strong anticoagulant, hemostasis, anti-inflammatory, and analgesic effects, while the SPN has better antioxidation and antiaplastic anemia activities [3–5]. However, the changes of content and type of saponins in the steaming process were responsible for the significant differences in the RPN and SPN. For example, with the prolongation of steaming time and the increase of temperature, the contents of notoginsenoside R_1_, ginsenoside Rg_1_, Re, Rb_1_, and Rd were gradually decreased, while the contents of ginsenosides Rh_1_, Rk_3_, Rh_4_, and Rg_3_ were gradually increased [3, 4]. The reason may be that due to the application of high temperature and pressure during the steaming process, the glycosidic bonds of saponins were cleaved and some of the glycosyl groups were removed, which may be accompanied with the dehydration reaction of the side chains and the configuration changes of C-20, and then converted into other saponins with less glycosyl groups [6].

Despite differences in pharmacological effects of RPN and SPN, there is no clear standard for the steaming of SPN. Until now, the processed PN has never been recognized by any pharmacopeias of different countries or regions [7–10]. Gu et al. [1] explored the effects of triterpenoids extracted from SPN which was steamed at 120°C for 10 h on the differentiation of PC12 cells. In addition, previous studies showed that most of the saponins in PN tend to be stable after 6 h of steaming at 120°C [4]. Moreover, the PN sample steamed at 120°C for 2 h was considered to be the best condition for extracting saponins by reverse phase extraction with ethanol [11]. Furthermore, there is also a lack of researches on the hematopoietic activities of SPN. Pharmacological experiments confirmed that Rk_3_, Rh_4_, and 20(*S*)-Rg_3_ have better antianemia effects [5, 12].

Moreover, different from the mechanism of chemical drugs, traditional Chinese medicine (TCM) has the characteristics of multicomponents, multitargeted, and multichannels, which emphasize integrity and systemicity [13]. It is difficult to reveal the scientific connotation of treating diseases by a single ingredient of TCM. With the development of multiomics theory and practice, the concept of network pharmacology came into being. Network pharmacy, as a method based on system biology, can analyze the relationship between drugs, targets, and diseases by predicting the complex interaction between the small molecules and proteins in biological systems, which is one of the effective methods to study the pharmacological effects of TCM [14–16]. Therefore, network pharmacology was used to predict and screen the active ingredients that contribute to the hematopoietic efficacy of SPN. As a predictive tool for the study of TCM, network pharmacology has uncertainties, so it is necessary to carry out pharmacological experiments on its results. In our study, pharmacological experiments *in vivo* were performed to verify the hematopoietic effects of the predicted active ingredients.

Furthermore, prevailing evidence demonstrated that Box-Behnken design response surface methodology (BBD-RSM) is widely used in the researches of the extraction, preparation, and processing of TCM, which is a set of mathematical and statistical techniques used to establish models for evaluating multiple parameters and their interactions with quantitative data and statistically and effectively optimizing the complex extraction procedures [11]. In the present study, we first used network pharmacology to predict the active ingredients related to the hematopoiesis effect of SPN and verified it by pharmacological experiments *in vivo*. Taking the total content of saponins with hematopoietic effect as the evaluation index, BBD-RSM was used to optimize the steaming conditions of SPN. We hope to provide and give methodology reference and a basis for the formulation of SPN steaming standard.

## 2. Materials and Methods

### 2.1. Plant Materials and Chemicals

SPN samples were obtained by steaming the RPN powder in an autoclave (Zhejiang, China) at different steaming conditions and then drying in an electric-thermostat-blast drying oven (Shanghai, China) at 40°C for 3 hours. The SPN samples were crushed and sieved through a 40-mesh sieve. Ginsenosides Re, Rd, Rh_1_, Rh_2_, Rk_3_, Rh_4_, 20(*S*)-Rg_3_, and 20(*R*)-Rg_3_ purchased from Chengdu Herbpurify Co., Ltd. (Sichuan, China) with purity ≥ 98% were used as standard compounds.

### 2.2. Animals

Specific-pathogen-free BALB/c mice were purchased from Guangdong Medical Laboratory Animal Center, China (SCXK(Yue)2018-0002), and kept in standard environment in the lab animal room in Clinical College, Hubei University of Chinese Medicine. The anemic model of mice was established according to the methods described by previous studies [12, 16]. In brief, BALB/c mice were injected with 360 mg/kg of RBV for 25 days. The reduction of HGB content to less than 60% of the control value was used as the criterion for judging the success of the modeling [16]. Before the experiments, the mice were given one-week acclimation period in the animal laboratory at room temperature (20-25°C) and constant humidity (40-70%) and fed with standard rodent feed and water freely. Animal experimental procedures in the study strictly conformed to the Guide for the Care and Use of Laboratory Animals and related ethics regulations of Hubei University of Chinese Medicine.

### 2.3. Screening the Blood-Enriching Constituents of SPN Using Network Pharmacology

#### 2.3.1. Database Constructing

Numerous studies have shown that saponins are the major active components of SPN, with pharmacologic effects such as blood enriching, antioxidation, antiplatelet, and anticoagulant [16, 17]. So, in our study, the twenty-four kinds of saponins of SPN were investigated including ginsenosides of CK, F_2_, F_4_, Rb_1_, Rb_2_, Rb_3_, Rc, Rd, Re, Rf, Rg_1_, Rg_5_, Rh_2_, Rh_4_, Rk_1_, Rk_3_, 20(*R*)-Rg_2_, 20(*S*)-Rg_2_, 20(*R*)-Rg_3_, 20(*S*)-Rg_3_, and Rh_1_ and notoginsenosides of C, R_1_, and R_2_ [5]. All of the chemical ingredients were obtained from literature research and Traditional Chinese Medicine Systems Pharmacology Database (TCMSP, http://lsp.nwu.edu.cn/tcmsp.php) [18, 19]. We searched the chemical structure of each compound in TCM Database@Taiwan (TD) (http://tcm.cmu.edu.tw/zh-tw/index.php), PubChem database (http://www.ncbi.nlm.nih.gov/pccompound), and Chemblink database (http://www.ebi.ac.uk/chembl) [18–20]. Then, the structure of the saponins was transformed into MOL2 format. In order to accurately predict the target proteins of SPN, the online target prediction software of PharmMapper (http://lilab.ecust.edu.cn/ pharmmapper; http://www.swisstarget prediction.ch/) with a criterion of ^“^fit score^”^ > 4 was used [5, 21]. And then, all the gene and protein targets associated with the disease of anemia were retrieved from Mendelian Inheritance in Man (OMIM) database to explore if they are related to candidate targets [22]. Meanwhile, the interaction of proteins and proteins plays an important role in signal transduction and regulation of gene expression. So, the database of Interacting Proteins (PPI) was employed to identify the possible interactions of the above-mentioned targets. And all protein ID codes were converted to UniProt IDs [23].

#### 2.3.2. Network Construction and Bioinformatics Analysis

To predict the hematopoietic active ingredients of SPN, we need to analyze the complex relationship between the constituents and targets associated with anemia. So, the “constituent-target-disease” network was constructed to help us study the blood-enriching mechanism. In the network, the nodes with different colors and shapes represented different compounds and targets, and the edges represented the interactions between the targets and compounds. Cytoscape 4.3 was used to visualize and analyze the network and calculate the topological features of each node in the network [24]. Only the hub nodes (twofold above the median “degree” value of all nodes) with higher values of “betweenness centrality” and “closeness centrality” (above the median value of all nodes) were identified as the candidate SPN targets for anemia [5]; the compound related to candidate SPN targets for anemia was considered as active ingredients for SPN.

### 2.4. Validation and Selection of the Screened Active Constituents

#### 2.4.1. Measurement of Blood Routine Parameters

To verify the hematopoiesis activities of the active constituents, mice were randomly divided into 18 groups (*n* = 10), namely, the control group, the model group, the *Fufang E'jiao Jiang* (FEJ) group, and the ginsenoside Rk_3_, Rh_4_, Rh_1_, 20(*S*)-Rg_3_, 20(*R*)-Rg_3_, Rd, Rh_2_, and Re low-, middle-, and high-dose groups (-L, -M, -H), respectively. After modeling, mice in the control group were administrated with 0.9% normal saline, whereas other groups were administrated with FEJ (8 mL/kg) and ginsenosides (25 mg/kg, 50 mg/kg, and 100 mg/kg) that lasted for 12 days, respectively. The blood was collected for the routine blood analysis after 30 min of the last administration. The levels of RBC, HCT, HGB, and PLT were detected by SE-9000 automatic blood instrument (Shanghai, China). Furthermore, liver and kidney indexes were recorded and analyzed.

#### 2.4.2. Detection of Hematopoietic Factor Expression

Trizol reagent (Invitrogen, USA) was used to extract total RNA from the serum of different groups in mice according to the manufacturer's instruction. Quantitative real-time PCR (qRT-PCR) analyses were performed with SYBR® Premix Ex Taq™ (Takara, USA). Relative quantification was performed using the 2^-*ΔΔ*CT^ method with GADPH as a housekeeping gene. The primers were synthesized by Invitrogen Company. The primer sequences were as follows: Kit ligand (Kitlg) forward primer 5′-TGGAGCTGCATGAAAAG TTG-3′ and reverse primer 5′-TCAGAGCAATCAATGCCAAG-3′; colony-stimulating factor 1 (Csf1) forward primer 5′-GCTCCTGCCTACCAAGACTG-3′ and reverse primer 5′-ATGGGCCATACAGGCTA GTG-3′; Csf3 forward primer 5′-CCTAGCAGGCATTTCCTCTG-3′ and reverse primer 5′-GGTGAGC TGTCTCCAGGAAG-3′; interleukin-6 (IL-6) forward primer 5′-TACCCCAACTTCCAATGCTC-3′ and reverse primer 5′-GGTTTGCCGAGTAGACCTCA-3′; interleukin-11 (IL-11) forward primer 5′-CTCCCCTC GAGTGTCTTCAG-3′ and reverse primer 5′-TCATGGCCAAGGTAGGTAGG-3′; leukemia inhibitory factor (Lif) forward primer 5′-CTCCCTGACCAACATCACCT-3′ and reverse primer 5′-GGACCACCGCACTAA TGACT-3′; and GADPH forward primer 5′-TGCCACTCAGAAGACTGTGGATG-3′ and reverse primer 5′-GCCTGCTTCACCACCTTCTTGAT-3′.

### 2.5. Optimization of Steaming Conditions of SPN by BBD-RSM Coupled with Hematopoietic Active Ingredients

#### 2.5.1. Steaming Process of SPN

In order to make the SPN have better blood-enriching effect, the active ingredients are used as evaluation indexes. And variables including the steaming time (2-10 h), steaming temperature (90-130°C), and producing area (Baoshan, Dali, Honghe, Lijiang, Puer, Qujing, Tengchong, Wenshan, and Yuxi) were investigated by using the BBD-RSM. A 500.0 g PN sample powder from different producing areas was separately placed in an autoclave (Zhejiang, China) and steamed at a given temperature and time. Subsequently, a 5.0 g sample powder of SPN was extracted by 50 mL of 70% ethanol for 1.5 h at 85°C in a water bath (Bang Xi Instrument Technology Co., Ltd. Shanghai, China). Repeat the extraction process three times, and the extract solution was combined, centrifuged, filtered, and concentrated to obtain the saponins of SPN which are related to hematopoietic effect.

#### 2.5.2. Determination of the Content of Saponins by HPLC Analysis

**Figure 1:**
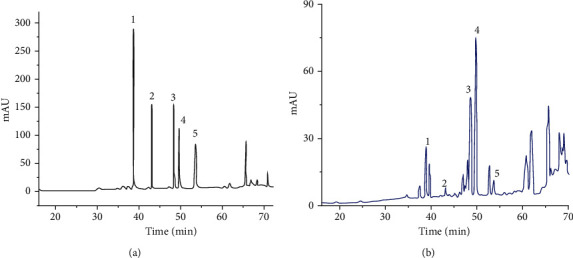
HPLC chromatograms of reference solution of (a) PN and (b) SPN samples (taken from the sample of SPN steamed for 5 h at 120°C as an example). Peaks 1, 2, 3, 4, and 5 correspond to ginsenosides Rh_1_, Rd, Rk_3_, Rh_4_, and 20(*S*)-Rg_3_, respectively.

The sample solutions were prepared by dissolving the crude saponins of SPN of 2.4.1 into a 20 mL volumetric flask and dissolving with pure water, shaken, filtered, and the subsequent filtrate reserved. The mixed standard solution was prepared by adding standard substances including Rd, Rh_1_, Rh_4_, Rk_3_, and 20(*S*)-Rg_3_ at 5 mg, respectively, into a 5 mL volumetric flask and dissolving with methanol. A series of standard working solutions with seven different concentrations were prepared by appropriately diluting the mixed stock solution with methanol for determination of the standard curves. The results of the HPLC analyses for the standard solution and sample solution were shown in Figures 1(a) and 1(b).

According to the published articles [5, 11], HPLC analysis was performed on an Agilent 1260 HPLC system (Agilent Technologies) consisting of a G1311B Pump, a G4212B DAD detector, and a G1329B autosampler. A Vision HT C18 column (250 mm × 4.6 mm, 5 *μ*m) was adopted for the analysis. The mobile phase was comprised of A (ultrapure water) and B (methyl cyanide). The gradient mode was as follows: 0-20 min, 80% A; 20-45 min, 54% A; 45-55 min, 45% A; 55-60 min, 45% A; 60-65 min, 100% B; 65-70 min, 80% A; and 70-90 min, 80% A. The flow rate was 1.0 mL/min. The detection wavelength was 203 nm. The column temperature was 30°C and sample volume was 10 *μ*L.

#### 2.5.3. BBD-RSM Experimental Design

The BBD-RSM was adopted to determine the optimal steaming conditions of SPN. And based on the results of the single-factor experiment analysis, we finally designed three-factor, three-level experiments involving steaming time (4-8 h), steaming temperature (110-130°C), and producing area (Honghe, Qujing, and Wenshan). The dependent variable (the content of saponins) was considered as the response. In this design, the test variable was transformed to the range of -1 to 1 for the evaluation of factors (Tables 1 and 2). The relationship between the three independent factors and the response was analyzed by Box-Behnken design via Design-Expert 8.0.6, including ANOVA, calculation of coefficients, and plotting. The general equation to predict the optimal point was explained as follows:

(1) $$\begin{array}{c} Y=\beta_{0}+\beta_{1}X_{1}+\beta_{2}X_{2}+\beta_{3}X_{3}+\beta_{11}X_{11}+\beta_{22}X_{22}+\beta_{33}X_{33}+\beta_{12}X_{12}+\beta_{13}X_{13}+\beta_{23}X_{23}, \end{array}$$

where *Y* is the predicted response, *β*_n_ represent the regression coefficients, and *X*_n_ are the coded independent factors. The regression coefficient (*R*^2^), adjusted *R*^2^ (*R*^2^_adj_), the prediction error sum of squares (PRESS), and adequate precision (AP) were used to determine the goodness of fit of the constructed polynomial models.

### 2.6. Determination of the Hematopoiesis Activity of the Optimal SPN

To determine the hematopoiesis activity of the optimal SPN, mice were randomly divided into six groups (*n* = 10), namely, the control group, the model group, the FEJ group, and the SPN at 120°C for 5 h low-, middle-, and high-dose groups (0.45 g/kg, 0.90 g/kg, and 1.8 g/kg), respectively, by gavage for 12 days. The blood was collected for the routine blood analysis after 30 min of the last administration. The levels of RBC, HCT, HGB, and PLT were detected by SE-9000 automatic blood instrument (Shanghai, China). Moreover, determination of the expressions of Kitlg, Csf1, Csf3, IL-6, IL-11, and Lif in different groups was done as discussed in Section 2.4.2.

### 2.7. Statistical Analysis

Results were obtained as the mean ± standard deviation. GraphPad Prism 5.0 software and SPSS 21.0 (Statistical Program for Social Sciences, Chicago, IN, USA) were used to perform Student's *t*-test and one-way ANOVA statistical analyses. *P* value < 0.05 was considered significant.

## 3. Results and Discussion

### 3.1. Screening the Blood-Enriching Constituents of SPN Using Network Pharmacology

#### 3.1.1. Prediction of the Compounds and Protein Targets

**Figure 2:**
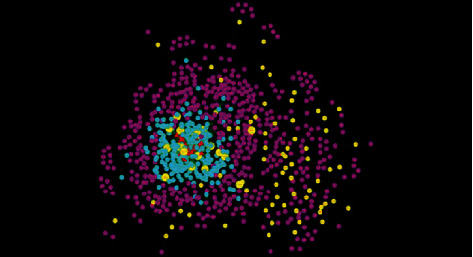
The “constituent-target-disease” pharmacology network of SPN saponins. The red triangles represented the saponins of SPN, the blue dots represented the indirect targets of those saponins, the yellow dots represented the targets of anemia, the purple dots represented the interactional proteins of the anemia targets and saponins of SPN, and the yellow squares represented the common targets of SPN saponins and anemia.

In our study, the twenty-four kinds of saponins of SPN which have been reported in the literature were investigated [2, 5, 25]. Based on the drug composition and protein target database, there were 810 protein targets involved in the construction of the pharmacological network of “constituent-target-disease,” including 205 indirect targets of those saponins, 87 targets related to anemia, and 518 interactional proteins associated with the anemia targets and SPN constituents (Figure 2). Among the above target proteins, 22 of which were the common targets of SPN constituents and anemia. Meanwhile, the common targets, as the directed targets of SPN on treating anemia, were relatively important for further screening. Therefore, we finally determined that the compounds with more association with these targets are the blood-enriching constituents.

#### 3.1.2. Network Construction and Bioinformatics Analysis

To predict the target proteins of SPN, the pharmacological network of “constituent-target-disease” was constructed. As shown in Figure 2, the network of the constituents and their potential targets was illustrated with color-coded nodes. The red triangles represented the constituents of SPN, the blue dots represented the indirect targets, the yellow dots represented the targets of anemia, the purple dots represented the interactional proteins of the anemia targets and SPN constituents, and the yellow squares represented the common targets of SPN constituents and anemia. As shown in Table 3, based on the network analysis by Cytoscape 4.3, eight saponins of SPN which directly connected with yellow squares were considered as our predicted ingredients.

### 3.2. Validation and Selection of the Screened Active Constituents *In Vivo*

**Figure 3:**
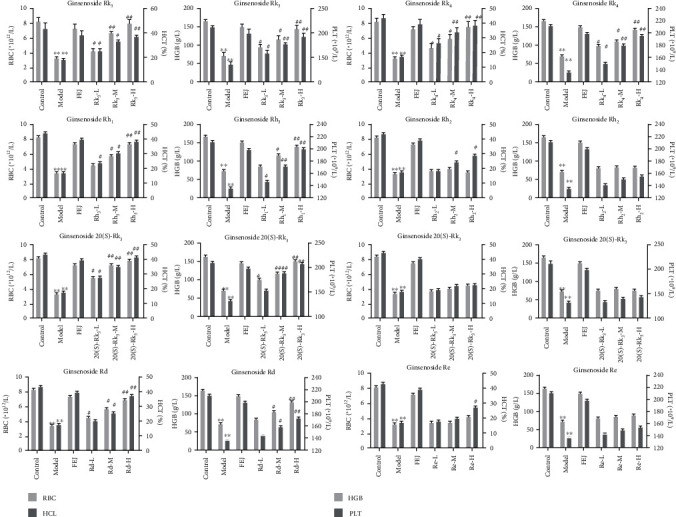
Effects of ginsenosides Rk_3_, Rh_4_, Rh_1_, Rh_2_, Re, Rd, 20(*S*)-Rg_3_, and 20(*R*)-Rg_3_ on the blood routine parameters in anemic mice. Data are expressed as mean ± standard deviation (*n* = 10). Model groups compared with the control groups, ^∗^*P* < 0.05, ^∗∗^*P* < 0.01. Ginsenoside groups compared with the model groups, ^#^*P* < 0.05, ^##^*P* < 0.01.

To further verify the hematopoiesis activities of ginsenosides Rk_3_, Rh_4_, Rh_1_, 20(*S*)-Rg_3_, 20(*R*)-Rg_3_, Rd, Rh_2_, and Re, the experiments *in viv*o were conducted. Anemia is characterized by the decrease of red blood cells (RBC), white blood cell specific volume (WBC), hemoglobin (HGB), and platelets (PLT) [26]. Previous studies demonstrated that ginsenosides Rk_3_ and 20(*S*)-Rg_3_ could significantly increase the levels of WBC, RBC, HGB, and PLT from the peripheral blood of mice [5]. Moreover, Wei et al. [12] found that ginsenosides Rk_3_ and Rh_4_ remarkably increased RBC, HGB, and red blood cell specific volume (HCT) and significantly improved the viscera and tissue injury in Ribavirin- (RBV-) induced anemia mice. In this study, the blood routine parameters were determined after administration. The results indicated that RBC, HCT, HGB, and PLT were significantly reduced in anemic mice, while obviously increased by ginsenosides Rk_3_, Rh_4_, Rh_1_, 20(*S*)-Rg_3_, and Rd in a dose-dependent manner (Figure 3). Furthermore, compared with the model groups, ginsenosides Rh_2_, Re, and 20(*R*)-Rg_3_ had no significant effect on the levels of RBC, HCT, HGB, and PLT. As shown in Table 4, compared with those in the control group, the liver and kidney indexes were significantly decreased in model mice. Moreover, compared with those in the model group, the liver and kidney indexes reduced by RBV were improved by ginsenosides Rk_3_, Rh_4_, Rh_1_, 20(*S*)-Rg_3_, and Rd in a dose-dependent manner. The results indicated that the liver and kidney of RBV-induced anemia mice were damaged, but this damage could be improved by ginsenosides Rk_3_, Rh_4_, Rh_1_, and 20(*S*)-Rg_3_.

**Figure 4:**
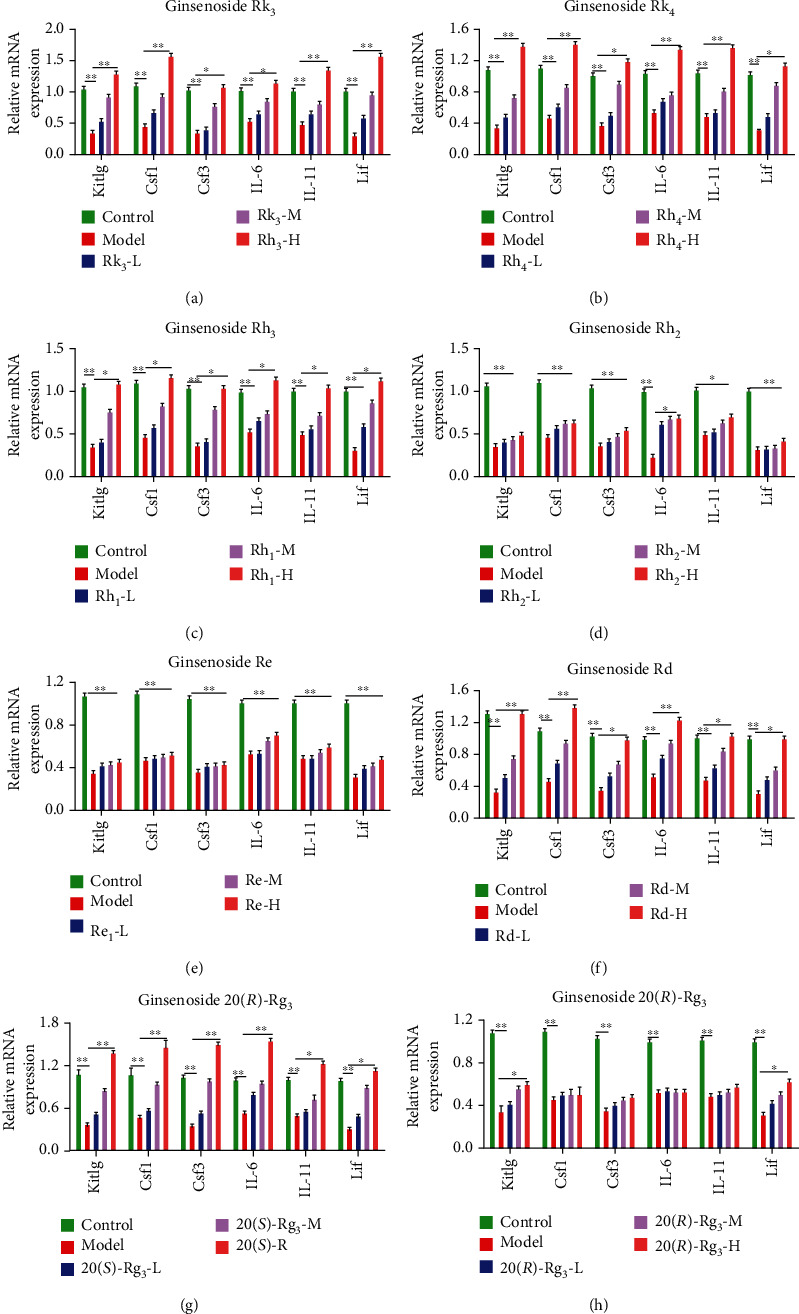
Effects of ginsenosides Rk_3_, Rh_4_, Rh_1_, Rh_2_, Re, Rd, 20(*S*)-Rg_3_, and 20(*R*)-Rg_3_ on the expressions of hematopoietic factors in anemic mice. Data are expressed as mean ± standard deviation (*n* = 5). Model groups compared with the control groups, ^∗^*P* < 0.05, ^∗∗^*P* < 0.01. Ginsenoside groups compared with the model groups, ^#^*P* < 0.05, ^##^*P* < 0.01.

Meanwhile, the expressions of Kitlg, Csf1,Csf3, IL-6, IL-11, and Lif in different groups were determined. Kitlg, also known as stem cell factor, is a factor that induces the growth of the earliest hematopoietic stem cells. It mainly stimulates the proliferation of hematopoietic stem/progenitor cells (HSC/HPC) and promotes the formation of hematopoietic colonies in coordination with various Csfs [27]. The main role of CSf1 (also called as M-CSF) is to promote the proliferation and differentiation of bone marrow HSC/HPC into monocyte colonies and induce the terminal differentiation of monocytes. Moreover, Csf1 could significantly improve the neuronal ischemic injury [28]. Csf3 (also called as G-CSF) mainly promotes the proliferation and differentiation of HSC/HPC into granulocyte colonies and induces the terminal differentiation of neutrophils [29]. The main functions of IL-3, IL-4, IL-6, and IL-11 are to support hematopoietic precursor cells in coordination with a variety of other cytokines, including promoting lymphocyte, myeloid, erythrocyte, and megakaryocyte cell lines to promote proliferation, differentiation, and maturation [30]. Moreover, Lif has a wide range of functions, which can inhibit the colony formation and induce differentiation of leukemia cells alone or in cooperation with IL-6 and Csfs [31]. In this study, compared with the control group, the expressions of Kitlg, Csf1,Csf3, IL-6, IL-11, and Lif were significantly decreased in the model group (Figure 4). Moreover, compared with those in the model group, the expressions of these hematopoietic factors reduced by RBV were improved by ginsenosides Rk_3_, Rh_4_, Rh_1_, 20(*S*)-Rg_3_, and Rd in a dose-dependent manner. The results also provide evidence for the changes of blood routine parameters in Figure 3. These results demonstrated that ginsenosides Rk_3_, Rh_4_, Rh_1_, 20(*S*)-Rg_3_, and Rd did have good hematopoietic function. Therefore, the best processing of SPN was explored by using the total content of ginsenosides Rk_3_, Rh_4_, Rh_1_, 20(*S*)-Rg_3_, and Rd as the evaluation index in the following experiments.

### 3.3. Optimization of Steaming Conditions of SPN by BBD-RSM Coupled with Active Constituents

#### 3.3.1. Effects of Steaming Temperature, Time, and Producing Areas on the Contents of SPN Saponins

**Figure 5:**
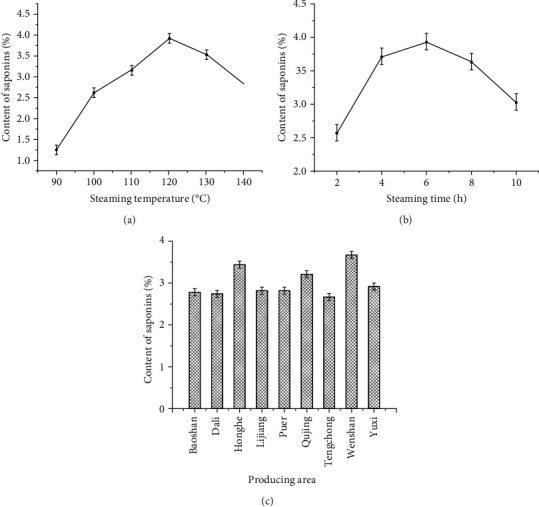
The results of single-factor experimental analysis.

Steaming temperature is one of the most important factors that could affect the content of saponins. The contents of saponins under different steaming temperature were shown in Figure 5(a). The results were obtained by, firstly, setting the steaming time and producing area to 6 h and Wenshan, respectively. The effect of steaming temperature on the content of saponins was then investigated by sequentially setting the steaming temperature at 90, 100, 110, 120, 130, and 140°C. According to the results, the content of saponins was firstly increased along with the steaming temperature, which took 120°C to reach the maximum. After that, the levels of the content of saponins decreased. The reason might be that at the initial stage of steaming, a relatively higher steaming temperature was beneficial for producing new saponins, such as Rh_4_, Rk_3_, and 20(*S*)-Rg_3_ [4]. However, if they were kept at high temperature for a long period of time, the original saponin components of PN could be easily decomposed, such as Rd and Rh_1_ [6]. Based on the results, the steaming temperature of 120°C could achieve saponins of higher content. Therefore, 110, 120, and 130°C were selected as optimal conditions in the following BBD-RSM experiments.

Another significant factor that would affect the content of saponins is the steaming time. Thus, in the present study, the effect of steaming time on the content of saponins was evaluated. The steaming temperature and the producing area were set as 120°C and Wenshan, respectively. The steaming time was then sequentially set as 2 h, 4 h, 6 h, 8 h, and 10 h. As shown in Figure 5(b), when the steaming time was varied between 2 h and 10 h, the content of saponins increased firstly and then decreased. The reason might be that at the initial stage of steaming, a relatively longer steaming time was beneficial for producing saponins, if they were kept at high temperature for a long period of time, triterpenoid saponins could be easily decomposed [6]. According to the results, the steaming times 4 h, 6 h, and 8 h were chosen to conduct experiments.

PN, a traditional and precious medicinal material unique to China, has special requirements for the environment. Its distribution is limited to the mid-high altitude area near 23°30′ of north latitude, including Yunnan and Guangxi [32]. In this study, we mainly investigated the effects of different producing areas of PN in Yunnan province on the content of saponins. As shown in Figure 5(c), under the same steaming conditions, the contents of saponins in Honghe, Qujing, and Wenshan were higher than those in other regions. So, we choose the above three places of origin as research objects in the following BBD-RSM experiments.

#### 3.3.2. Fitting the Response Surface Models

As shown in Tables 1 and 2, based on single-factor experimental analysis, three-factor, three-level experiments involving steaming temperature (*X*_1_), steaming time (*X*_2_), and producing area (*X*_3_) were designed in the BBD-RSM experiment. The experiments were performed by Box-Behnken design via Design-Expert 8.0.6 software (Stat-Ease Inc., Minneapolis, MN, USA), and the content of saponins under different conditions was shown in Table 2. According to the experimental data, the second-order polynomial equation was applied to express the content of saponins of SPN as a function of variables.

The content of saponins = 3.93 + 0.27 *X*_1_ − 0.10 *X*_2_ − 0.041 *X*_3_ − 0.13 *X*_1_*X*_2_ − 0.094 *X*_1_*X*_3_ + (2.250E − 003) *X*_2_*X*_3_ − 0.58*X*_1_^2^ − 0.16*X*_2_^2^ − (2.475*E* − 003)*X*_3_^2^.

**Figure 6:**
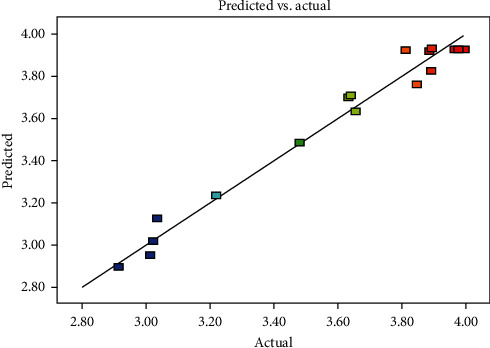
The comparison between the predicted and measured values of the content of the saponins from the SPN.

The analysis of variance (ANOVA) was used to estimate the statistical significance of factors and their interaction [11]. For each term in the models, a large *F* value and a small *P* value would imply a more significant effect on the respective response variable. As shown in Table 5, the model was highly significant (*P* < 0.0001), and the lack of fit was not significant (*P* = 0.2434 > 0.05), indicating the model was able to predict the content of saponins under any combination of values of the variables. To verify the adequacy of a model, the coefficient of determination (*R*^2^), the adjusted determination coefficient (*R*^2^_adj_), AP, and CV tests were calculated. In our model, the *R*^2^ value was 0.9759, indicating that the experimental data were very consistent with the second-order polynomial equation and only 2.41% of the total variation which was not explained by the models. The *R*^2^_adj_ value of 0.9449 was in reasonable agreement with the value of the *R*^2^ values, indicating that the model was significant. The AP value of 14.822 was greater than 4, indicating that this model could be used to navigate the design space. As a general rule, the CV should not be greater than 10%, and in our model, the CV value of 2.55 was desirable. Moreover, Figure 6, where predicted versus actual values are plotted, also verifies the suitability of the model; the predicted value of the model and the actual value of the experiment were fitted almost in a straight line. It proved that the second-order polynomial regression model was in good agreement with the experimental results and indicated that the models applied in this study were able to identify the operating conditions for selective steaming conditions of SPN.

#### 3.3.3. Response Surface Test Results and Analysis

In this model, as shown in Table 5, the liner and quadratic effects of the steaming temperature and steaming time were significant (*P* < 0.05), indicating that the two factors had a highly significant effect on the content of saponins. However, the *P* value of places of origin was 0.2408 (*P* > 0.05), indicating that the producing area (Wenshan, Honghe, and Qujing) had no significant effect on the content of saponins. Meanwhile, it could be seen from the coefficients of the regression equation that the steaming time had the greatest influence on the response value of the content of saponins, followed by steaming temperature and places of origin (*X*_2_ > *X*_1_ > *X*_3_). Among the different interaction effects, there was only one interaction of steaming temperature (*X*_1_) with steaming time (*X*_2_), which was significant (*P* < 0.05).

**Figure 7:**
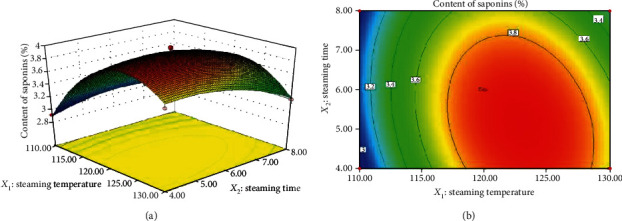
Response (a) surface and (b) contour plots showing the significant (*P* < 0.05) interaction effects of steaming temperature with steaming time on the content of saponins.

Figure 7(a) presents the interaction between the steaming temperature and steaming time. The content of saponins was initially increased along with the duration of the steaming, following the decrease which might be due to the fact that it takes a certain amount of time for the original saponin component in PN to be converted into a new saponin [3, 4]. At the same time, the content of saponins was significantly decreased by increasing the steaming temperature, which was probably due to the fact that the temperature promotes the conversion of saponin. The higher the temperature, the better the conversion of the new ingredients of PN [33]. However, if the temperature is too high, it will destroy the saponin of PN and reduce the content of saponin [34]. As shown in Figures 7(a) and 7(b), the content of the saponins reached the maximum when the steaming temperature and steaming time were approximately 130°C and 60%, respectively.

#### 3.3.4. Optimization and Validation Procedures

In order to make the SPN have a better blood-enriching effect, the steaming conditions of PN need to be optimized to obtain the maximum saponin content which is associated with blood-enriching effects. So, the quadratic models were established, and the predicted maximum value (3.99%) of the test variables was found under the following conditions: steaming time 5 h, steaming temperature 120°C, and place of origin Wenshan. Experiments were performed under the recommended optimum steaming conditions; the actual values of the content of saponins were 4.02% ± 0.05% (*n* = 3), which were very close to the values predicted by the RSM models. Xiong et al. [4] have examined the content of SPN under different steaming conditions, and their experimental results are consistent with ours.

### 3.4. The Hematopoiesis Effects of the Most Optimal SPN *In Vivo*

**Figure 8:**
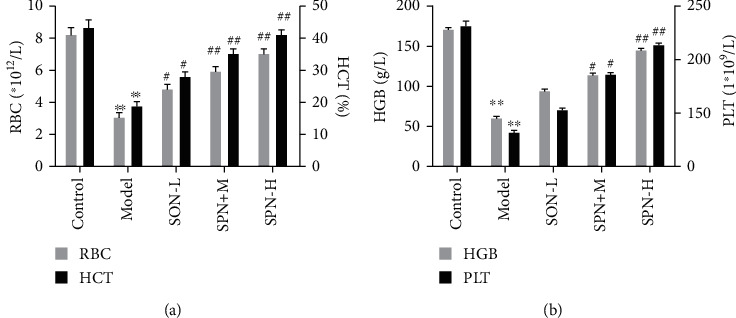
The hematopoiesis effects of SPN steamed at 120°C for 5 h evaluated by peripheral blood parameter measurement. Data were expressed as mean ± standard deviation (*n* = 10). The model groups compared with the control groups, ∗*P* < 0.05, ∗∗*P* < 0.01. The SPN groups compared with the model groups, ^#^*P* < 0.05, ^##^*P* < 0.01.

**Figure 9:**
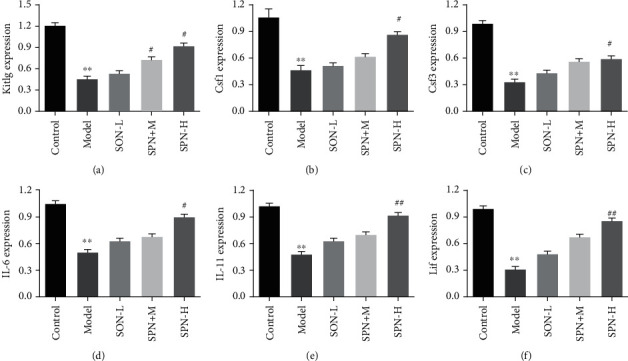
The effects of SPN steamed at 120°C for 5 h on hematopoietic factor expressions. Data were expressed as mean ± standard deviation (*n* = 5). The model groups compared with the control groups, ∗*P* < 0.05, ∗∗*P* < 0.01. The SPN groups compared with the model groups, ^#^*P* < 0.05, ^##^*P* < 0.01.

As shown in Figures 8(a) and 8(b), the quantities of RBC, HCT, HGB, and PLT from the peripheral blood of mice were detected after the end of the administration of SPN powder. Compared with the model group, SPN significantly increased the levels of RBC, HCT, HGB, and PLT in a dose-dependent manner. The results were similar to the previous study [16]. Compared with the model group, SPN alleviated the shrinking of hepatic nucleus and cytoplasm vacuole degeneration. Furthermore, we also explored the effects of the most optimal SPN on the expressions of hematopoietic factors. As shown in Figure 9, results showed that high dose of SPN significantly increased the expressions of Kitlg, Csf1,Csf3, IL-6, IL-11, and Lif. The results demonstrated that the most optimal SPN had better hematopoiesis effects.

## 4. Conclusions

In the research, to obtain a kind of SPN which has better blood-enriching effect, the bioactive constituents of SPN were unveiled and verified by network pharmacology coupled with pharmacology experiments. Results showed that Rd, Rh_1_, Rh_4_, Rk_3_, and 20(*S*)-Rg_3_ were the active constituents related to hematinic. Meanwhile, the effect of steaming time, steaming temperature, and producing area on the total content of the five saponins from SPN was studied and successfully optimized by using BBD-RSM, respectively. The optimal steaming conditions were as follows: steaming time 5 h, steaming temperature 120°C, and producing area Wenshan. Furthermore, the optimal SPN could significantly increase the levels of blood routine parameters, hematopoietic factors compared with the model group. The study not only provides data support for the determination of hematinic effect-related markers for SPN but also gives a scientific reference for the processing of SPN which has a better hematopoietic effect.

## Figures and Tables

**Table 1:** Experimental domain of BBD-RSM.

Independent variables	Unit	Symbol	Coded levels		
			-1	0	+1
Steaming temperature	°C	*X*_1_	110	120	130
Steaming time	h	*X*_2_	4	6	8
Producing area		*X*_3_	Honghe	Wenshan	Qujing

**Table 2:** The BBD matrix and the experimental data for the responses.

Treatment No.	Steaming temperature (°C)	Steaming time (h)	Producing area	Content of saponins (%)
1	-1	0	1	3.037
2	0	-1	-1	3.889
3	1	1	0	3.214
4	0	1	-1	3.643
5	1	-1	0	3.635
6	-1	-1	0	2.914
7	0	0	0	3.896
8	-1	1	0	3.015
9	-1	0	-1	3.025
10	0	1	1	3.658
11	0	-1	1	3.895
12	0	0	0	3.992
13	0	0	0	3.979
14	0	0	0	3.812
15	1	0	1	3.482
16	0	0	0	3.967
17	1	0	-1	3.846

**Table 3:** The active constituents and the corresponding common target proteins predicted by network pharmacology analyses.

Constituent	Structure	Target proteins (UniProt no.)
Ginsenoside Rd	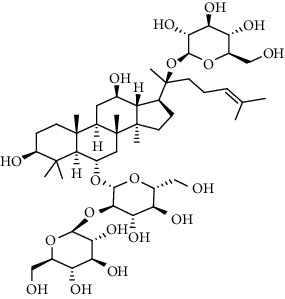	P16442 P06702 Q06124
Ginsenoside Re	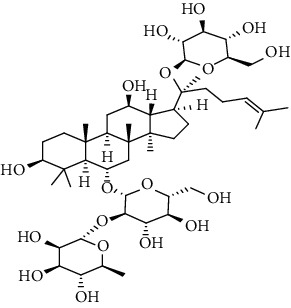	P16442 P30613
Ginsenoside Rh_2_	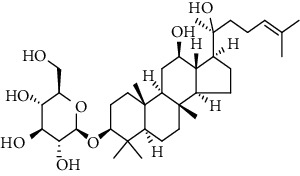	P16224
Ginsenoside Rh_4_	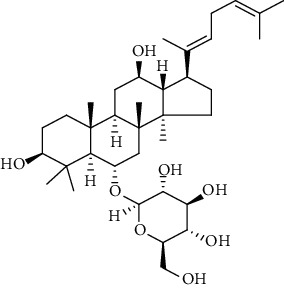	P16442 P06702 Q06124
Ginsenoside Rh_1_	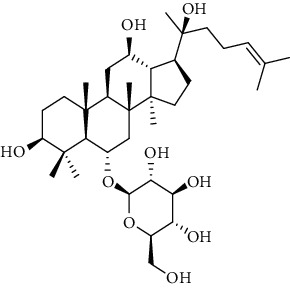	P16442 P06702
Ginsenoside Rk_3_	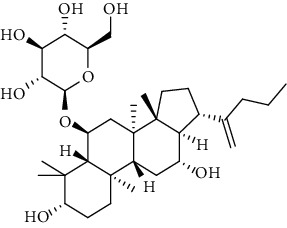	P22830 P16442 P06702 Q06124
Ginsenoside 20(*S*)-Rg_3_	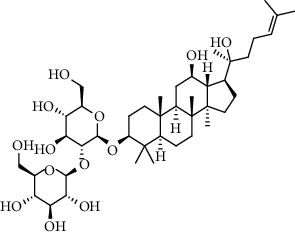	P22830 P16442
Ginsenoside 20(*R*)-Rg_3_	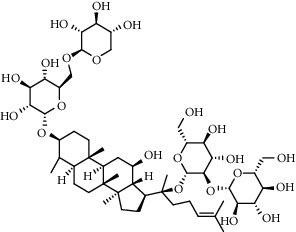	P16442

Remark: P16442: histo-blood group ABO system transferase; P06702: protein S100-A9; Q06124: tyrosine-protein phosphatase nonreceptor type 11; P30163: pyruvate kinase PKLR; P22830: FECH, mitochondrial.

**Table 4:** Effects of the active constituents responsible for SPN hematopoietic function on visceral index in mice (*n* = 10).

Grouping	Liver index (mg/g)	Kidney index (mg/g)
Control	56.55 ± 4.21	11.95 ± 1.81
Model	33.19 ± 2.48^∗∗^	5.48 ± 0.82^∗∗^
Rk_3_-L	37.23 ± 3.21	7.08 ± 1.01^#^
Rk_3_-M	43.58 ± 4.11^#^	9.23 ± 1.32^##^
Rk_3_-H	48.72 ± 5.40^##^	10.58 ± 1.65^##^
Rh_4_-L	39.22 ± 2.95^#^	7.85 ± 0.98^#^
Rh_4_-M	45.37 ± 4.10^##^	9.05 ± 1.54^##^
Rh_4_-H	47.23 ± 4.58^##^	9.89 ± 1.68^##^
Rh_1_-L	36.84 ± 3.98	6.85 ± 1.47
Rh_1_-M	40.57 ± 4.09^#^	8.29 ± 1.09^#^
Rh_1_-H	43.87 ± 3.79^#^	9.23 ± 0.74^##^
20(*S*)-Rg_3_-L	41.82 ± 4.35^#^	7.91 ± 0.98^#^
20(*S*)-Rg_3_-M	46.08 ± 3.38^##^	9.02 ± 1.33^##^
20(*S*)-Rg_3_-H	49.21 ± 1.24^##^	10.66 ± 1.58^##^
Rd-L	39.44 ± 3.89	7.54 ± 1.61^#^
Rd-M	46.21 ± 5.01^##^	8.39 ± 0.94^##^
Rd-H	47.85 ± 4.58^##^	9.09 ± 1.01^##^

Note: model groups compared with the control groups, ∗*P* < 0.05, ∗∗*P* < 0.01. Ginsenoside groups compared with the model groups, ^#^*P* < 0.05, ^##^*P* < 0.01.

**Table 5:** The ANOVA results for the response surface quadratic models for the content of saponins of SPN.

Source.(content of saponins)	Sum of squares	DF	Mean square	*F* value	*P* value
Model	2.36	9	0.26	31.47	<0.0001
*X*_1_	0.6	1	0.6	71.64	<0.0001
*X*_2_	0.081	1	0.081	9.67	0.0171
*X*_3_	0.014	1	0.014	1.64	0.2408
*X*_12_	0.068	1	0.068	8.17	0.0244
*X*_13_	0.035	1	0.035	4.24	0.0785
*X*_23_	2.03*E* − 05	1	2.03*E* − 05	2.43*E* − 03	0.9621
*X*_1_^2^	1.41	1	1.41	169.42	<0.0001
*X*_2_^2^	0.1	1	0.1	12.21	0.0101
*X*_3_^2^	2.58*E* − 05	1	2.58*E* − 05	3.09*E* − 03	0.9572
Residual	0.058	7	8.34*E* − 03		
Lack of fit	0.036	3	0.012	2.1	0.2434
Pure error	0.023	4	5.67*E* − 03		
Cor total	2.42	16			
*R* ^2^	0.9759				
*R* ^2^_adj_	0.9449				
CV	2.55				
AP	14.822				

## Data Availability

All data used to support the findings of this study are available from the corresponding author upon request.

## Abbreviations

AP: Adequate precision
BBD-RSM: Box-Behnken design response surface methodology
FEJ: *Fufang E'jiao Jiang*
HGB: Hemoglobin
HCT: Red blood cell specific volume
PLT: Platelet
PN: *Panax notoginseng*
PRESS: Prediction error sum of squares
*R*^2^: Regression coefficient
RBC: Red blood cell
RBV: Ribavirin
RPN: Raw PN
SPN: Steamed *Panax notoginseng*
TCM: Traditional Chinese medicine
WBC: White blood cell
Kitlg: Kit ligand
Csf: Colony-stimulating factor
IL-6: Interleukin-6
IL-11: Interleukin-11
Lif: Leukemia inhibitory factor
HSC/HPC: Hematopoietic stem/progenitor cells.
